# Development of a loop-mediated isothermal amplification assay for detection of *Austropeplea tomentosa* from environmental water samples

**DOI:** 10.1186/s44149-022-00061-9

**Published:** 2022-12-12

**Authors:** Lily Tran, Vignesh A. Rathinasamy, Travis Beddoe

**Affiliations:** 1 Department of Animal, Plant and Soil Sciences, School of Agriculture, Biomedicine and Environment, La Trobe University, Bundoora, VIC 3083 Australia; 2 Australian Institute of Tropical Health and Medicine and Queensland Tropical Health Alliance, James Cook University, Cairns, QLD 4870 Australia

**Keywords:** *Fasciola* spp., Snail, Molecular detection, DNA diagnostics, LAMP, Environmental sampling, eDNA

## Abstract

Lymnaeid snails are key intermediate hosts for the development and survival of *Fasciola* spp., the causative agent of Fascioliasis which are economically important parasites infecting humans and livestock globally. The current control method for treating Fascioliasis is heavily reliant on anthelmintic drugs, particularly Triclabendazole (TCBZ) which has resulted in drug-resistant parasites and poses significant risk as there are no long-term efficacious alternatives available. Sustainable control measures at the farm level could include both parasite and snail control will play an important role in *Fasciola* spp. control and reduce the reliance on anthelmintic drugs. Implementation of such sustainable control measures requires effective identification of snails on the property however Lymnaeid snails are small and difficult to physically locate. Snail identification using an environmental DNA approach is a recent approach in which physically locating snails are not required. *Austropeplea tomentosa,* is the primary intermediate snail host for *F. hepatica* transmission in South-East Australia and we present an in-field loop-mediated isothermal amplification and water filtering method for the detection of *A. tomentosa* eDNA from water samples to improve current surveillance methods. This methodology is highly sensitive with a detection limit of 5 × 10^− 6^ ng/μL, detected in < 20 minutes, with cumulative sample preparation and amplification time under 1 hour. This proposed workflow could assist in monitoring areas to determine the risk of Fascioliasis infection and implement strategies to manage snail populations to ultimately reduce the risk of infection for humans and livestock.

**Supplementary Information:**

The online version contains supplementary material available at 10.1186/s44149-022-00061-9.

## Introduction

Fascioliasis imposes a substantial economic burden to agricultural livestock sector globally and human health problems in developing countries. The causative agent of this zoonotic disease are the liver flukes *Fasciola hepatica* commonly found in temperate regions, and *F. gigantica* found in tropical regions. Combined, these flukes have a global distribution and are endemic in three continents, with fasciolosis considered a neglected tropical disease of developing countries with estimates of 2 – 17 million people infected globally (Ai et al. 2010; Boray et al. 1969; González et al. 2011). Infection results in irreversible liver damage, reducing production yields in livestock, namely sheep, goat, and cattle, where human infection frequently results in gastrointestinal complications, including cholangitis (Young et al. 2012). The mainstay treatment for fasciolosis is triclabendazole (TCBZ), the only drug lethal to the juvenile and adult stages, however multiple reports of TCBZ-resistant (TCBZ-R) flukes have emerged in Europe, Australia, and South America, including human cases (Brockwell et al. 2014; Kelley et al. 2016).

With multiple reports of TCBZ-R flukes, a reduction on anthelmintic reliance is urgently required. The options of drug-mediated control of liver flukes are generally restricted for human cases as TCBZ is the only approved anthelmintic for human fasciolosis, and is compounded by waning efficacy due to resistance concerns in livestock (Hien et al. 2008). Furthermore, regions where the access to drugs is limited due to economic restrictions, lack of commercial access and supply are in urgent need of alternative control measures that are effective, sustainable, and easily deployable to manage liver fluke infection. Therefore, alternative measures should be considered without reliance on costly anthelmintics.

One alternative is interrupting the *Fasciola* spp. lifecycle and preventing infection instead of disease treatment. *Fasciola* spp. development is reliant on freshwater snails where liver fluke eggs are excreted from infected host faeces into the environment, embryonate in water and hatch into miracidium. These seek out a snail intermediate host of the *Lymnea* genus, with *Austropeplea tomentosa* the key species in South-East Australia and undergoes development into sporocysts. Rediae then as free-swimming cercariae which encyst on vegetation as metacercariae, the infective stage for the definitive host being ungulates or humans to ingest. Metacercariae then excyst in the duodenum as juveniles migrating through the host liver for 6-8 weeks until maturation, thereafter residing in the biliary ducts producing eggs (Young et al. 2010). Clearly intermediate snail hosts are essential for fluke survival, hence disrupting or minimising the presence of Lymnaeid snails in the environment would reduce the likelihood of lifecycle completion and therefore infection (Mas-Coma et al. 2009).

Lymnaeid snails are freshwater snails, many of which serve as intermediate hosts for several trematode species including *Echinostoma* spp., *Schistosoma* spp., and *Paramphistomum* spp., in addition to *Fasciola* spp. (Correa et al. 2010; Soldánová et al. 2010). They generally inhabit shallow freshwater environments, including rivers, puddles and water ditches (Bui et al. 2016), and are the intermediate host for multiple helminth species. For example, *Galba truncatula*, a native European Lymnaeid with distribution in South America and Asia, is the intermediate host for both *Fasciola* spp. and *Calicophoron daubneyi* (Jones et al. 2018).

Despite the epidemiological importance of snails in trematode prevalence, snail surveys are overwhelmingly conducted using physical identification (Rathinasamy et al. 2018). This generally requires copious amounts of time and labour, with snail identification ambiguous and difficult to determine genus let alone species level identification, requiring molecular identification (Bargues et al. 2001). The integral role of snails in trematode development highlights the importance of improving snail survey methods, as assessing snail prevalence can identify areas with highest snail abundance and therefore infection risk. This understanding can assist in the mitigation of *Fasciola* spp. transmission.

Although improved snail detection methods are required, few molecular studies have been published. Of these, the majority is performed performed using PCR-based amplification including conventional and quantitative (qPCR) methods for either snail species identification and/or *Fasciola* spp. detection from whole snail preparations, necessitating the collection of physical specimens (Alba et al. 2015; Cucher et al. 2006; Magalhães et al. 2008; Magalhães et al. 2004). Recent studies have seen the development of PCR assays for the detection of environmental DNA (eDNA) from trematodes and/or their intermediate hosts (Hashizume et al. 2017; Hung and Remais 2008; Jones et al. 2018; Rathinasamy et al. 2021).

eDNA is DNA shed by the oraganism into the surrounding environment, hence physically locating specimens is not essential (Lugg et al. 2018). Molecular eDNA surveys show greater sensitivity, reporting positive detection of target species in the absence of physical specimens and is frequently used for the surveillance of endangered and/or invasive species (Belle et al. 2019; Turner et al. 2014). However, these methods require costly filtering equipment, logistics and a well-equipped laboratory with trained staff. Further, eDNA is unstable and rapidly deteriorirates, often at the time of sampling, requiring extensive cold-chain transport, or stabilisation buffers to minimise eDNA degradation (Renshaw et al. 2015).

As fasciolosis commonly occurs on rural or remote areas where access and transport to and from facilities is difficult, current eDNA methods to the best of our knowledge are not suitable for in-field use of molecular eDNA surveys. Consequently molecular eDNA surveys should be shifted for in-field detection for use in remote regions, and to minimise the effects of rapid eDNA degradation. One way to achieve this is through the use of loop-mediated isothermal amplification (LAMP), a rapid DNA amplification method amplifying DNA in under an hour. Amplification is conducted between 60- 65°C through a strand displacing polymerase which does not require a thermocycler enabling reactions to be incubated in a waterbath, heatblock or through a portable fluorometer for real-time detection (Amoah et al. 2017; Njiru 2012; Notomi et al. 2000). Furthermore, the strand displacing polymerases used in LAMP assays are generally less suceptible to inhibition of DNA amplification (Nagamine et al. 2001). Consequently, LAMP assays present as a suitable method for in-field eDNA surveys for targets of interest using crude DNA extraction methods without reliance on laboratory processing.

To improve current snail detection methods and provide a new and easily deployable tool to detect liver fluke-transmitting snails in farms, we have developed an *Austropeplea tomentosa* specific LAMP assay (with an eDNA isolation method from water samples) that is suitable for field-use. This workflow enables in-field eDNA surveys of *A. tomentosa*, the key intermediate snail host for *F. hepatica* in South-East Australia, and can be used to assist in mitigating liver fluke infection risk on Victorian farming properties, with further applicability to remote regions where Fascioliasis is a concern.

## Results

### Determining optimal *Austropeplea tomentosa* LAMP primers

Three *A. tomentosa* LAMP primer (AtLAMP) sets were designed in this study due to high sequence similarity with Lymnaeid snails. As LAMP primers necessitate the use of inner primers (FIP&BIP) which are generally 30-40 nt long each, in conjunction with a suggested product length for the F3 and B3 primers of 200-300 bp for optimal loop formation, this further limited primer design due to the partial ITS-2 sequence of *A. tomentosa* being 413 bp. Consequently, it was not possible to design primers specific to *A. tomentosa* LAMP in silico*,* requiring the design of multiple primer sets to assess which set yielded the fastest T_p_ and least cross-reactivity. As non-target amplification was observed for all primer sets used (Table 1), optional loop primers were individually assessed to see if this increased T_p_ values of non-target snails whilst retaining optimal T_p_ values for *A. tomentosa* as T_a_ values of non-target snails were near identical to *A. tomentosa* values returning T_a_ values between 93 and 93.8°C for all non-target snails. Consequently, secondary discrimination through T_a_ analysis was not feasible.

**Table 1:** *A. tomentosa* LAMP primers and primer combinations assessed in this study to reduce non-target snail species amplification

Primers and snails assessed	Primer combination used and average T_**p**_ values (mm:ss)			
**Primer set: 1**	**LF + LB**	**LB**	**LF**	**No loops**
Pa	10:73	17:73	15:80	–
Pc	11:08	18:30	14:73	–
Gt	11:15	18:65	15:23	–
Al	12:00	19:65	16:23	–
Av	8:15	10:38	11:73	18:80
At	6:00	9:45	8:00	17:38
**Primer set: 2**	**LF + LB**	**LB**	**LF**	**No loops**^a^
Pa	9:30	11:30	10:73	13:23
Pc	9:65	11:73	11:08	14:00
Gt	9:45	11:38	11:15	13:73
Al	10:30	12:73	11:15	14:45
Av	9:15	11:45	10:15	13:38
At	4:45	6:00	5:30	6:73
**Primer set: 3**	**LF + LB**	**LB**	**LF**	**No loops**
Pa	17:04	22:73	22:15	Not assessed
Pc	17:34	23:30	22:73	Not assessed
Gt	17:15	23:80	22:80	Not assessed
Al	18:51	25:50	24:38	Not assessed
Av	17:61	26:23	24:15	Not assessed
At	9:69	14:15	12:38	Not assessed

^a^denotes chosen primer combination used throughout study

All primer combinations used for each *A. tomentosa* LAMP primer set, including the addition of both loops (LF&LB), one loop (LB or LF), and no loops were assessed using a starting concentration of 5 × 10^0^ ng/μL synthetic DNA for each snail species (Table 1). No loop primers (LF and LB) were not assessed for the third primer set as removing one loop primer greatly increased *A. tomentosa* T_P_’s to > 12 minutes, therefore negatively impacting the likelihood of detection at lower starting DNA quantities.

As all primers recorded T_p_ values for non-target snails with near identical T_a_’s, it was therefore necessary to select the primer set that yielded the lowest T_p_ for *A. tomentosa*, balanced with the greatest T_p_ difference from non-target snails. The isothermal mastermix used in this study (ISO-DR004, OptiGene) recommends an amplification cut-off time of 20 minutes, therefore T_p_ values for non-target snails were first assessed for T_p_’s close to, or exceeding 20 minutes (set 1: LB, LF, no loops, set 2: no loops, set 3: LF + LB, LB, LF). Secondly, *A. tomentosa* T_p_’s were analysed to assess T_p_ values with some combinations (set 1: no loops and set 3, all combinations) considered too slow for reliable amplification of low DNA starting qualities and were subsequently excluded, as T_p_’s were close to or exceeding 10 minutes. Therefore two options remained (set 1 with LF and set 2 no loops), with the second set selected for use throughout this study providing lower T_p_’s for *A. tomentosa* detection (average T_p_: 6:73, compared to 8:00 for set 1 with LF) in addition to providing the greatest differentiation of non-target snail amplification, with T_p_ differences of 65-73% slower amplification compared to 37-68% for set 1.

### Analytical performance of the *Austropeplea tomentosa* LAMP

Assay performance of AtLAMP was determined using a ten-fold serial dilution of *A. tomentosa* standards with starting concentrations ranging from 5 × 10^0^ to 5 × 10^− 6^ ng/μL. Amplification times ranged from 06:45 – 20:00 (mm:ss), and assay analytical sensitivity determined to be 5 × 10^− 6^ ng/μL (Table 2), with samples at this concentration returning an average T_p_ of 19:08, all replicates consistently amplifying through all 10 runs, further demonstrated by low inter-assay variation of < 3% (Table 2). No difference in T_p_ values was observed when inner primer concentrations were increased from 1.6 to 1.8 or 2 μM, with starting DNA concentrations of 5 × 10^− 1^ ng/μL amplifying between 7:30-7:45, consistent with 1.6 μM inner primer concentrations (Fig. S1). As there was no reduction in T_p_ values, 1.6 μM was chosen as the optimal primer concentration.

**Table 2:** Inter-assay co-efficient of variation of *A. tomentosa* gDNA serial dilutions using LAMP

Starting DNA concentration (ng/μL)	Average T_p_ (mm:ss)	Inter-assay CV (%)
5 × 10^0^	6:98 ± 0.24 SD	3.48
5 × 10^−1^	8:42 ± 0.24 SD	2.87
5 × 10^−2^	10:55 ± 0.32 SD	2.99
5 × 10^−3^	12:59 ± 0.41 SD	3.24
5 × 10^−4^	14:34 ± 0.29 SD	2.00
5 × 10^−5^	16:40 ± 0.41 SD	2.52
5 × 10^−6^	19:08 ± 0.52 SD	2.74

**Fig. 1:**
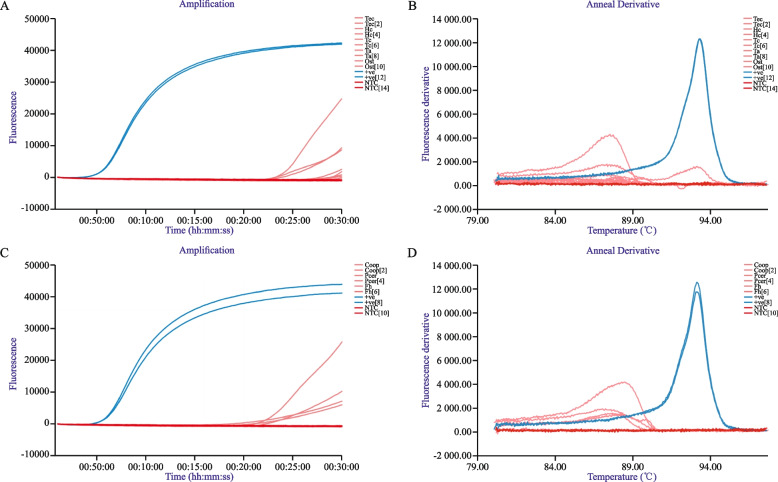
Specificity panel used to determine AtLAMP specificity using common livestock helminths and *F. hepatica.***A**,**C** LAMP amplification curves for AtLAMP performed on DNA from common livestock helminths and *F. hepatica* performed in duplicate (Tec - *Teladorsagia circumcincta;* Hc - *Haemonchus contortus;* Tc- *Trichostrongylus* colubriformis; Ta-*T. axei*; Ost- *Ostertagia* spp.; Coop- *Cooperia* spp.; Pcer-*Paramphistomum cervi*; Fh-*F. hepatica; +ve-snail DNA;* NTC-No template control*).* T*. axei* and *Cooperia* spp. recorded one of two replicates with T_p_ values exceeding 25 minutes, with T_p_ values from *H. contortus, Ostertagia* spp., and *F. hepatica* exceeding 29 minutes from one of two replicates. **B**,**D** The fluorescent derivative annealing curve (T_a_) for the corresponding livestock helminths and *F. hepatica* DNA. No T_a_ values recorded for all helminth species assessed

The specificity of AtLAMP from non-target snails was determined through primer selection described earlier and was further determined using gDNA from common livestock helminths (Table 1). Samples were considered negative if T_p_ values were not consistently recorded and no T_a_ observed. The specificity panel using helminth gDNA returned intermittent T_p_ values exceeding 25 minutes from one of two duplicate replicates for *T. axei,* and *Cooperia* spp., with *H. contortus, Ostertagia* spp., and *F. hepatica* yielding T_p_’s exceeding 29 minutes from duplicate replicate samples (Fig. 1 A, C). No corresponding T_a_ values were observed, suggesting high assay specificity from mixed samples (Fig. 1 B, D).

### Quantitative PCR analysis of *Austropeplea tomentosa* detection from environmental DNA samples

AtLAMP performance using environmental water samples was confirmed using a previously established eDNA isolation method with minor modifications.

The modified eDNA isolation method described demonstrates high eDNA recovery from environmental water samples, with DNA recovery rates using this method exceeding 100% and low Cq values (< 15, Table 3). This may be due to larger volumes used to spike DNA relative to qPCR template volumes used being 2.5 μL, thus increased spiking volumes may have resulted in the underestimation of back calculated starting quantities, and/or from NanoDrop™ over quantifying actual DNA abundance.

**Table 3:** Moat water samples spiked with varying starting quantities of *A. tomentosa* DNA for environmental DNA isolation methods, estimated final concentrations, and their respective average Cq values and SD, used to calculate actual final concentrations of DNA in each sample used to assess DNA recovery from each method used

Sample starting quantity (μg)	Estimated final concentration (ng/μL)	Average Cq	Calculated final concentration (ng/μL)	Recovery (%)
**NaOAC + NaI clean up**				
1	9.9 × 10^−2^	+	N/A	–
0.5	5 × 10^−2^	+	N/A	–
0.25	2.5 × 10^−2^	13.53 ± 0.21 SD	7.73 × 10^−2^	> 100
0.125	1.25 × 10^−2^	12.49 ± 0.68 SD	1.56 × 10^−1^	> 100
Neg	–	ND	–	–
**Filtering – 50 mL vol**				
1	2 × 10^−^^2^	15.93 ± 0.18 SD	1.53 × 10^−^^2^	76.7
0.5	1 × 10^−^^2^	17.52 ± 0.31 SD	5.25 × 10^−^^3^	52.54
0.25	5 × 10^−^^3^	18.37 ± 0.10 SD	2.92 × 10^−^^3^	58.47
0.125	2.5 × 10^−^^3^	19.53 ± 0.05 SD	1.36 × 10^−^^3^	54.24
Neg	–	ND	–	–
**Filtering – 250 mL vol**				
1	8.5 × 10^−^^3^	16.88 ± 0.08 SD	8.09 × 10^−^^3^	95.15
0.5	4.25 × 10^−^^3^	18.38 ± 0.11 SD	2.94 × 10^−^^3^	69.25
0.25	2.13 × 10^−^^3^	20.35 ± 0.01 SD	7.80 × 10^−^^4^	36.72
0.125	1.06 × 10^−^^3^	21.36 ± 0.30 SD	3.95 × 10^−^^4^	37.19
Neg	–	ND	–	–

A crude eDNA isolation method was trialled with all water samples processed using the Sterivex® filter successfully amplifying irrespective of total volume filtered. These samples returned Cq values ranging between 15.50-21.36 (± < 0.5 SD) across all spiked samples (Table 3). Expectedly, Cq values obtained from the filtering method irrespective of volume and starting quantity were higher in comparison to the modified eDNA method due to the lack of subsequent concentration and clean up steps. Regardless, filtering drastically reduced sample processing time and cost from at least 1 hr. to 15 minutes for five samples, whilst eliminating sodium iodide use. Further, DNA recovery rates from spiked samples ranged between 52 and 76% from 50 mL volumes of each spiked sample (Table 3) with the lowest calculated final concentration determined as 1.36 × 10^− 3^ ng/μL from an initially estimated starting concentration of 2.5 × 10^− 3^ ng/μL *A. tomentosa* DNA. Increasing filtering volume to 250 mL whilst retaining the same starting DNA quantities resulted in increased Cq values ranging from 16 to 22 Cq (± < 0.5 SD) in comparison to lesser filtrate volume, with varying DNA recovery rates between 37 and 95%. The lowest final concentration from these samples was calculated as 3.95 × 10^− 4^ ng/μL from an estimated starting concentration of 1.06 × 10^− 3^ ng/μL of spiked *A. tomentosa* DNA. Irrespective of volume filtered, the qAt assay successfully amplified all samples prepared using the extraction free filtering method.

In addition, no amplification was observed in the un-spiked samples suggesting high specificity from highly heterogenous samples.

### Loop-mediated isothermal amplification of *Austropeplea tomentosa* from environmental DNA samples

**Fig. 2:**
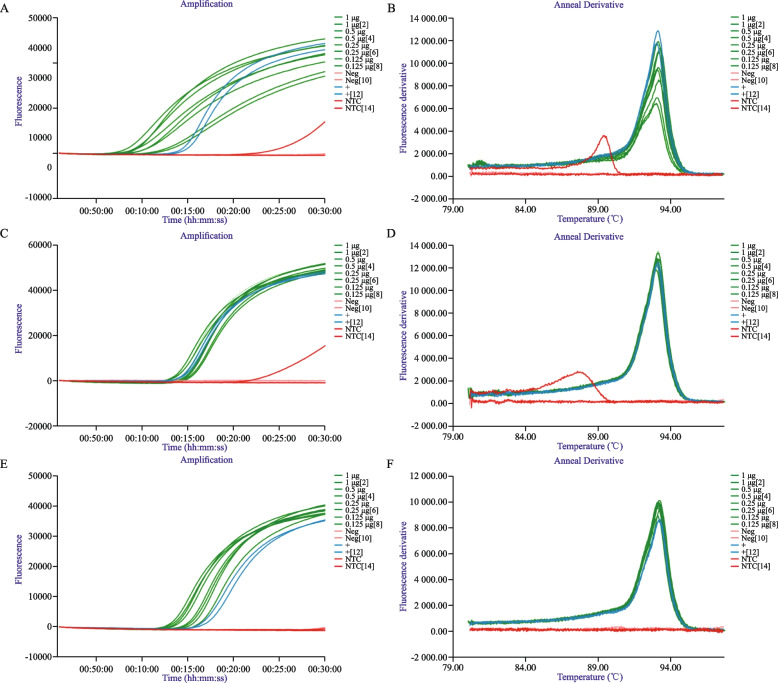
AtLAMP performed on different *Austropeplea tomentosa* water environmental DNA isolation methods. LAMP amplification curves for AtLAMP performed on range spiked *A. tomentosa* DNA amounts purified by ethanol precipitation and silica dioxide purification (**A**), 50 mL by filtration (**C**) and 250 mL by filtration (**E**). The fluorescent derivative annealing curve for the corresponding ethanol precipitation and silica dioxide purification (**B**), 50 mL by filtration (**D**) and 250 mL by filtration (**F**)

As both eDNA methods were successful using the q-PCR assay (qAt), samples were further assessed using AtLAMP to compare detection rates. Firstly the modified eDNA method was used to confirm sample suitability with all samples recording T_p_ values ranging from 10 to 15:45 mm:ss (Fig. 2). Thereafter the filtering method was analysed with T_p_ values from all spiked samples ranging from 15 to 16:45 mm:ss from 50 mL filtrate and 14-17 mm:ss from 250 mL volumes. All filtered samples were successfully detected, with 100% detection of *A. tomentosa* DNA from spiked samples and no observed amplification from the un-spiked moat water (Table 4).

**Table 4:** Average time-to-positive (Tp, mm:ss) values of water samples spiked with varying quantities of *A. tomentosa* by LAMP

	Water eDNA purification method		
DNA starting quantity (μg)	NaOAC + NaI clean up	Filtering 50 mL vol	Filtering 250 mL vol
1	10:73 ± 1.03 SD	15.15 ± 0.21 SD	14:80 ± 0.49 SD
0.5	11:80 ± 0.92 SD	15.35 ± 0.11 SD	14:80 ± 0.49 SD
0.25	13:80 ± 2.33 SD	16.23 ± 0.32 SD	16:45 ± 0 SD
0.125	12:80 ± 3.32 SD	16.30 ± 0.21 SD	17:58 ± 0.60 SD
0	ND	ND	ND

*ND* not detected

Although amplification times were slower in filtered samples, T_p_’s irrespective of initial sample starting volume were still < 20 minutes, with slower amplification not unexpected consistent with qPCR data obtained. Given all filtered samples were successfully detected using AtLAMP, this suggests that a simple, crude filter capture method may be suitable for *A. tomentosa* detection from environmental water samples, returning T_p_ values < 20 minutes with high specificity, failing to amplify off-target species from un-spiked moat water samples without the need for laboratory and transport facilities.

## Discussion

Fasciolosis is an economically important parasitic disease of livestock and a neglected tropical disease in humans. Current treatment methods have limitations due to increasing reports of TCBZ-R flukes, with further limitations on long term management options. Commercial vaccines are not available to control liver fluke infection and experimental vaccines have shown varying protection levels with further research required to develop effective liver fluke vaccines (Toet et al. 2014). Consequently, reliance on TCBZ or other anthelmintics must be reduced and sustainable alternative methods to mitigate parasite prevalence would ease the reliance on anthelmintics. One such alternative is the surveillance of the intermediate snails capable of transmitting liver flukes and implement snail control programs to minimise the risk of liver fluke infection in livestock or humans (Boray 1964).

Shifting management practices from treatment to infection prevention through targeting the snail intermediate host would greatly reduce *F. hepatica* prevalence in the environment and add to the limited arsenal of existing liver fluke control measures, where Boray determined that increased Lymnaeid snail numbers correlated to increased risk of fasciolosis in naïve tracer sheep on an irrigated dairy property, further reinforcing the importance of Lymnaeid snails in trematode development (Boray et al. 1969).

Despite recent developments in *Fasciola* spp. detection, few advancements have been published in the literature for Lymnaeid snails required in the *Fasciola* spp. lifecycle, with most studies focusing on *Schistosoma* spp. intermediate snail hosts (Gandasegui et al. 2016; Kumagai et al. 2010; Mesquita et al. 2021; Tong et al. 2015; Velusamy et al. 2004; Yuan et al. 2005). Though these studies largely focus on the detection of schistosome DNA within the snail intermediate host, which still necessitates physically finding snails. Physically searching for snails is a tedious and erroneous process, often resulting in false negative reporting of snail detection due to their small size, and are frequently missed in the field due to surveying large areas, making it impossible to survey an entire paddock (Jones et al. 2018).

Recently qPCR assays were developed for trematode detection using eDNA from water sources to remove the requirement of physically searching for trematodes or their intermediate hosts (Hashizume et al. 2017; Rathinasamy et al. 2021). Despite these improvements, qPCR assays are costly and require specialised laboratory equipment and facilities for successful eDNA isolation and amplification. An additional constraint is eDNA transport from the field to a laboratory which generally involves transport from rural or remote areas and requires cold-chain transport to minimise eDNA degradation as eDNA is unstable with reports of deterioration at the time of collection, hence cold-chain transport or stabilising buffers are essential to minimise degradation (Renshaw et al. 2015).

To mitigate issues of sample logistics and rapid eDNA degradation, a proof-of-concept for a field-suitable method of snail (*A. tomentosa*) detection from environmental water samples were presented in this research. We have selected *A. tomentosa* in the proof-of-concept assay as it is the primary liver fluke transmitting snail species in South-East Australia (Boray 1964). The methodology presented here of filtering to concentrate free DNA could detect as little as 3.95 × 10^− 4^ ng/μL *A. tomentosa* DNA in less than 20 minutes, with total time to results from sample preparation and amplification complete in less than 1 h. Previous *A. tomentosa* eDNA concentrations from a Victorian dairy farm studied in Rathinasamy (Rathinasamy et al. 2021) reported concentrations ranging from 3.58 × 10^− 1^ – 3.2 × 10^− 4^ ng/μL *A tomentosa* eDNA (average eDNA concentration: 2.08 × 10^− 2^ ng/μL, IQR2: 5.56 × 10^− 3^ ng/μL) over 7 collection periods. Considering the detection limit of AtLAMP reported here as 5 × 10^− 6^ ng/μL, with the average quantity of *A. tomentosa* DNA detected from previous studies being 2.08 × 10^− 2^ ng/μL, assuming a 30% DNA recovery rate from 250 mL filtrate demonstrated in this study, average eDNA levels would be approximately 1.45 × 10^− 2^ ng/μL, with these eDNA concentrations falling within the AtLAMP limit of detection. The high sensitivity of AtLAMP is likely attributed to the target gene being the internal transcribed spacer 2 sequence being purposely selected. This gene is commonly used for DNA diagnostics as it is present in high abundance in species genomes occurring as tandem repeats, thereby increasing the likelihood of detection, thus resulting in high assay sensitivity (Bargues et al. 2001; Le et al. 2008).

Thus comparing *A. tomentosa* detection between both qAt and AtLAMP assays, total assay run time is reduced from 60 minutes to < 40 minutes including sample preparation and reaction set-up. This methodology is mobile and can be transported and use outside laboratory settings due to the Genie II fluorometer portability from an internal battery and availability of lyophilised LAMP reagents (Bath et al. 2020). Combined with high sensitivity (5 × 10^− 6^ ng/μL), comparable to the established qAt, this suggests that AtLAMP has the potential for molecular point of care testing without dedicated thermocyclers needed for PCR-based assays whilst retaining diagnostic performance.

To the best of our knowledge to date, no assays have been published for in-field molecular eDNA surveillance of trematode transmitting snails from water samples. In addition to in-field applications, the assay presented here has high specificity with no non-target amplification observed from the un-spiked moat water samples, known to contain a variety of invertebrates including gastropods (Gooderham and Tsyrlin 2002). Further, the careful selection of primers enables discrimination from non-target snails based on T_p_’s, resulting in > 50% slower amplification from non-target snails, assessed using a high starting concentration of synthetic standards. Therefore non-target snail amplification is not expected from water sampling due snail DNA being shed at low concentrations in the environment (Thomas et al. 2019). Alternatively, primers used in this study could be modified for the addition of degenerate bases and instead used for the universal detection of *Fasciola* spp. transmitting snails including *Pseudosuccinea columella, G. truncatula,* and *A. viridis* in regions where fluke transmitting snails overlap (Bui et al. 2016; Molloy and Anderson 2006)*.*

The snail detection has been critical in the successful management of schistosome infections. Emphasis on snail detection and disrupting snail habitats have seen reductions in schistosome infections, with successful eradication of *Schistosoma japonicum* in Japan using such measures (Tanaka and Tsuji 1997). The development of a field-suitable molecular eDNA surveillance method presented here can be modified for the detection of key trematodes of interest, which share similar waterborne development stages. For example, pre-existing LAMP assays for schistosome and small liver fluke detection reported in Gandasegui et al. (2015); Gandasegui et al. (2018); Le et al. (2012); Mesquita et al. (2021); Mwangi et al. (2018); Xu et al. (2010) can be adapted for use with the water eDNA sampling method described here to establish low-cost parasite surveillance programs from trematode or intermediate host eDNA detection. Consistent eDNA detection can be a strong indicator of prevalence and enable identification of areas known to harbour snails and therefore water-reliant parasite development stages such as shallow water bodies, including puddles and riverbanks. By prioritising areas with frequent eDNA detection, eDNA surveys can be conducted to monitor snail or parasite presence without having to physically locate specimens, enabling informed management actions to reduce high risk infection areas.

## Conclusion

This is the first report of a proof-of-concept approach to in-field eDNA surveys for *A. tomentosa.* The AtLAMP and eDNA filtration method presented here demonstrate high sensitivity and rapid turnaround, reporting results in under 1 h inclusive of amplification time. The assay requires further validation with water samples collected from known areas/farms where fasciolosis has been identified and it can be readily used in large-scale testing of field collected water samples after such validation. This methodology can be used for the surveillance of snail eDNA and therefore fasciolosis risk through monitoring *A. tomentosa* eDNA positive sites, which may be used in management decisions to mitigate parasite prevalence. Further, this assay and workflow has the potential to be used for identification of other snail species with minor primer modification and aid in the universal detection of liver fluke transmitting snails in areas where fasciolosis is a neglected tropical disease and anthelmintic treatment is not affordable/feasible. Identifying areas with positive snail eDNA detection can assist in reducing the risk of infection or enable prioritisation for snail control practices to reduce populations and therefore reduce the infection risk to humans and animals.

## Methods

### Snail standards synthetic DNA preparation for assay optimisation

Sample acquisition was not feasible for all snail species used in this study that either serve as the intermediate host for *F. hepatica* or share the same ecology as *F. hepatica* transmitting snails in South-East Australia. *Galba truncatula* is exotic to Australia, however, was included as this is a key intermediate host in Europe and South America and used as an internal positive control in previous studies (Rathinasamy et al. 2018; Rathinasamy et al. 2021). Synthetic DNA constructs containing the internal transcribed spacer (ITS-2) region of commonly found snails in Australia, in addition to *Galba truncatula* endemic to Europe (Table 5) were cloned into pBHA vectors by Bioneer (Daejeon, South Korea) and transformed into chemically competent XL1 Blue *E. coli* (New England BioLabs, Massachusetts, United States) following manufacturer instructions. Total plasmid DNA was extracted using the AccuPrep® Plasmid Mini Extraction Kit (Bioneer, Daejeon, South Korea) as per manufacturer instructions and plasmid DNA was eluted twice with TE buffer (10 mM Tris-HCl, 0.1 mM EDTA pH (8.4)) pre-heated to 65°C. Samples were assessed for quality through NanoDrop™ Spectrophotometer (ThermoFisher Scientific, Massachusetts, USA) and stored at −20°C until needed.

**Table 5:** Common snails found in Australia used for DNA extraction for specificity panel. Synthetic constructs containing partial ITS-2 insert of snails and corresponding amplicon size used in this study

Species	Abbreviation	GenBank accession	pBHA vector (bp)	ITS-2 insert (bp)	Total amplicon size (bp)	Relevance
*Physa acuta*	Pa	KF316328.1	257	302	559	Common in South-East Australia, non-fluke transmitting
*Pseudosuccinea columella*	Pc	HQ283261.1		535	792	Common in NSW, northern VIC, fluke transmitting
*Galba truncatula*	Gt	KT280457.1		496	753	Not in Australia, fluke transmitting
*Austropeplea lessoni*	Al	EU556308.1		486	743	Common in Southern and Eastern Australia, non-fluke transmitting
*A. viridis*	Av	EU556313.1		397	654	Common in central NSW, southern QLD, fluke transmitting
*A. tomentosa*	At	EU556270.1		411	668	Common in South-East Australia, key fluke transmitting species in VIC

Due to the pBHA vector backbone being 1987 bp, it was necessary to truncate the vector sequence to minimise interference for strand displacing polymerase amplification. Synthetic plasmid constructs containing the partial ITS-2 sequence of snails (Table 5) were PCR amplified to produce amplicons of varying sizes (Table 5). Amplification was carried out in 50 μL reaction volumes comprising of 1x *Pfu* DNA polymerase buffer with MgSO_4_ (Promega, Madison, USA), 200 μM each dNTPs, 0.4 μM each pBHA primers (pBHA_F 5′-3′: ATTGTCTCATGAGCGGATAC, pBHA_R 3′-5′: GCGTTATCCCCTGATTCTGT (Bioneer, Daejeon, South Korea)), 1.25 U *Pfu* DNA polymerase (Promega, USA), 0.1 μg synthetic construct and adjusted to a final volume of 50 μL with nuclease free water (NFW).

Amplification was performed using the following parameters: initial denaturation at 95°C for 2 min, follow by 30 cycles of denaturation at 95°C for 30 sec, annealing at 60°C for 30 sec and extension at 72°C for 45 sec, followed by a final extension at 72°C for 5 min. PCR products were separated on a 2% (*w*/*v*) agarose gel prepared with 0.5 x tris-borate EDTA buffer, stained with 0.5 μg/mL ethidium bromide and visualised using a Gel Doc EZ system (Bio-Rad, California, USA). Amplicon clean-up was performed using a FastGene Gel/PCR Extraction Kit (NIPPON Genetics, Tokyo, Japan) following manufacturer’s instructions and eluted twice in TE buffer. DNA yield was quantified using Qubit dsDNA BR (ThermoFisher Scientific, USA). Sequences were confirmed by Sanger Sequencing performed at the Australian Genome Research Facility (Victoria, Australia) and stored at −20°C until required.

### *Austropeplea tomentosa* LAMP primer design

Loop-mediated isothermal amplification primers for *A. tomentosa* detection were designed to target the ITS-2 sequence described earlier. Primers (Table 6) were designed using either Primer Explorer V5 (Eiken Chemical Company; https://primerexplorer.jp/e/) with default settings or CLC Genomics (Qiagen, Hilden, Germany) to manually design LAMP primers following primer design requirements as described by Nagamine and Notomi (Nagamine et al. 2002; Notomi et al. 2000).

**Table 6:** Loop-mediated isothermal amplification primers used for *Austropeplea tomentosa* detection in this study

Primer name	Primer sequence 5′-3′^a^	Sequence region 5′-3′
Set 1 F3	GTCTCAAGCACAAGCCGC	F3
Set 1 B3	GGGCGCCGATTTGTCAAG	B3
Set 1 FIP	GAGGAAAATTTGGCCGCCGCCCGTTGTCCGTGTTCGTC	F1,F2
Set 1 BIP	CTAACGGGCCCGCTCGTAACTCAGCGTAAGCTTCTCTCCT	B1, B2
Set 1 LF	AGAGCAAGGCGGCGT	LF
Set 1 LB	AAGCTCCAGGGTGATTGCG	LB
Set 2 F3^a^	TGCACGGTGTTGCCCG	F3
Set 2 B3^a^	GCGTAAGCTTCTCTCCTCCG	B3
Set 2 FIP^a^	GCGGACGTCCCGAGACGAACTGGCCCCGTGGTCTCAA	F1,F2
Set 2 BIP^a^	TTCCTCCTCGTCACCGCTATGCCAATCACCCTGGAGCTTGTT	B1, B2
Set 2 LF	GCGGCGGCCAAATT	LF
Set 2 LB	CGGCTCGCTCTCGCTAA	LB
Set 3 F3	GGTGGCCCCGTGGTCT	F3
Set 3 B3	CTTCTTCAATTCGTACGGGCG	B3
Set 3 FIP	AGCGGTGACGAGGAGGAAAATTCGTGTTCGTCTCGGGACGT	F1,F2
Set 3 BIP	CGTAACAAGCTCCAGGGTGATTGTTGTCAAGCGAGCGTCAGC	B1, B2
Set 3 LF	CGAGAGCAAGGCGGCGT	LF
Set 3 LB	CGGAGGAGAGAAGCTTAC	LB

^a^denotes chosen primer combination used throughout study

### *Austropeplea tomentosa* LAMP

*Austropeplea tomentosa* LAMP (AtLAMP) primers (Table 6) were assessed for lowest amplification time. Final amplification conditions were optimised in 25 μL reaction volumes comprising of 15 μL isothermal mastermix (ISO-DR004, OptiGene, Horsham, United Kingdom), 5 μL primer mix with optimised final concentrations of 1.6 μM inner primers (FIP&BIP), 0.2 μM outer primers (F3&B3), and 5 μL template per manufacturer instructions. During initial validation assessing all three primer sets (Table 7), primer concentrations consisted of 1.6 μM inner primers, 0.2 μM outer primers, and 0.4 μM loop primers (LF&LB). All LAMP runs included a positive control of *A. tomentosa* standards prepared as described earlier to monitor inter-assay variation in addition to NTC reactions using NFW to monitor contamination.

**Table 7:** Common livestock helminths and life stages used for DNA extraction for specificity panel

Species	Abbreviation	Life stage
*Teladorsagia circumcincta*	Tec	Adult
*Haemonchus contortus*	Hc	Adult
*Trichostrongylus colubriformis*	Tc	L3
*T. axei*	Ta	L3
*Ostertagia* spp.	Ost	L3
*Cooperia* spp.	Coop	L3
*Paramphistomum cervi*	Pcer	L3
*Fasciola hepatica*	Fh	Adult

Isothermal amplification was performed using a Genie II fluorometer (OptiGene) with an initial pre-heat of 40°C, 1 min, followed by amplification at 65°C, 30 min, and anneal from 94 to 84°C at 0.5°C/second. Amplification was recorded as time to positive (T_p_) in minutes and seconds (mm:ss), with anneal derivative melting temperature (T_a_) reported in degrees Celsius (°C). Assay performance was determined by assessing the analytical sensitivity and inter-assay variation of AtLAMP by performing 10 replicate runs using a ten-fold serial dilution of *A. tomentosa* standards with starting concentrations of 5 × 10^0^ – 5 × 10^− 6^ ng/μL performed in duplicate. Assay limit of detection was determined where 95% of samples recorded positive T_p_ values with coefficient of variation below 10%, with assay analytical specificity assessed using prepared snail and helminth DNA.

### *Austropeplea tomentosa* quantitative PCR

Quantitative PCR was used as a reference assay to validate and compare AtLAMP results. An *A. tomentosa* specific quantitative PCR (qAt) was modified from Rathinasamy et al. (2018) and performed as a singleplex for *A. tomentosa* detection. Amplification was performed in 25 μL volumes containing 1x SensiMix II Probe mastermix (Bioline, Alexandra, Australia), 0.3 μM each forward (5′-GCCAAATTTTCCTCCTCGT-3′) and reverse (5′- AAGCGAGCGTCAGCGTAA-3′) primer, 0.15 μM probe (HEX-CTAACGGGCCCGCTCGTAACA-BHQ-1), 1 mM MgCl_2_ to obtain a final concentration of 4 mM MgCl_2_, and 2.5 μL sample diluted 1/10 per sample. Amplification was carried out in a MIC qPCR cycler (BioMolecular Systems, Queensland, Australia), using a two-step cycling method as follows: 90°C, 10 min, followed by 40 cycles of 95°C, 10 sec and 60°C, 20 sec. All qPCR runs included a positive control of *A. tomentosa* standards to monitor intra-assay variation, and NTC reactions with NFW to assess contamination. Data analysis was performed using the MIC PCR program (V2.6.4), using the dynamic normalisation method, a defined threshold of 0.5 normalised fluorescence units, with the first amplification cycles excluded.

### Helminth genomic DNA preparation for specificity test

Helminths infecting ruminant that are commonly found in livestock in South-East Australia (Table 7) were kindly provided by Dr. Tim Elliot (Invetus, Armidale, New South Wales). Samples were washed three times using 70% EtOH prior to genomic DNA (gDNA) extraction. Between 20 and 25 mg of adult helminth tissue was excised from the anterior end and mechanically disrupted using scalpel blades before being transferred to microfuge tubes. As weighing larvae was not feasible, L3 larvae provided in 50 mL conical tubes containing 70% EtOH were centrifuged at 13000 x *g* at 4°C for 15 min to form a pellet. Samples were checked for a visible pellet, excess EtOH removed, and larvae transferred to a microfuge tube using a transfer pipette. Total gDNA was obtained per Brindley et al. (1989), with minor modifications as follows: 25 μL grinding buffer containing 80% *v*/*v* homogenization buffer (100 mM NaCl, 200 mM sucrose, 10 mM EDTA, 30 mM Tris-HCl pH 8.0 and 20% *v*/*v* lysis buffer (250 mM EDTA pH 8.0, 2.5% *w*/*v* SDS, 500 mM Tris-HCl pH 9.2), pre-heated to 60°C was added to each tube containing each helminth and were further mechanically disrupted using pellet pestles (Merck, New Jersey, United States). Samples were then incubated 65°C for 30 min before adding 14 μL 8 M ammonium acetate for a final concentration of 1 M, before incubation on ice for 30 min to precipitate proteins, then pelleted by centrifugation at 4°C, 13000 x *g* for 10 min. The resulting supernatant containing DNA transferred to a fresh microfuge tube, discarding the protein pellet for standard ethanol precipitation.

Ethanol precipitation was performed through the addition of 2.5 volumes chilled absolute ethanol and 1/10 vol 3 M sodium acetate (NaOAc) to each sample. Tubes were inverted and incubated at −20°C for 60 min, or overnight before pelleting precipitated DNA by centrifugation at 4°C, 13000 x *g* for 15 min. Samples were assessed for visible pellets before removing the ethanol supernatant. DNA pellets were then washed with chilled 70% ethanol and centrifuged to re-pellet DNA before discarding the ethanol wash. Samples were washed a minimum three times in 70% ethanol before elution. Prior to elution, tubes were inverted and air dried to evaporate residual ethanol before the addition of 50 μL TE buffer pre-heated to 65°C and pipetted to dissolve the DNA pellet. DNA quality was estimated through a NanoDrop™ 2000 Spectrophotometer with A260/280 and A260/230 values between 1.8-2.0 considered acceptable quality for DNA amplification. Purified helminth gDNA was then standardised to a final concentration of 5 ng/μL in TE buffer and stored at −20°C until needed.

### Environmental DNA isolation

Water known to be free from *A. tomentosa* and *F. hepatica* was collected from the La Trobe University (LTU) moat (GPS coordinates: −37.718, 145.044). Samples were spiked with known starting quantities consisting of 1, 0.5, 0.25 and 0.125 μg of *A. tomentosa* synthetic DNA prepared as described earlier. Due to ongoing COVID-19 travel restrictions, access to known *A. tomentosa* positive farming properties was not possible. As eDNA is unstable, transport logistics could not guarantee sample integrity either, therefore artificial spiking was used.

Environmental DNA isolation was initially performed as described in Rathinasamy et al. (2018), however, we have modified the protocol with reduced sodium iodide (NaI) quantity to reduce running costs and environmental impact. This method was initially used to determine the suitability of AtLAMP for *A. tomentosa* detection using water environmental samples. Briefly, LTU moat water samples were aliquoted into 10 mL volumes and spiked with various starting quantities of *A. tomentosa* DNA consisting of 1, 0.5, 0.25, and 0.125 μg. Final concentrations of *A. tomentosa* DNA in each spiked sample were back calculated to estimate concentrations of 9.9 × 10^− 2^_,_ 5 × 10^− 2^_,_ 2.5 × 10^− 2^ and 1.25 × 10^− 2^ ng/μL for each spiked sample respectively. A negative extraction control consisting of un-spiked LTU moat water was included to assess non-target amplification.

Samples were mixed by inversion then subjected to ethanol precipitation described earlier with minor modifications: samples were pelleted by centrifugation at 4700 x *g* for 40 min, ethanol supernatant discarded, and the resulting DNA pellets resuspended in 500 μL TE to ensure samples were fully dissolved due to larger pellet size. As environmental water samples contain numerous organic inhibitors which are often co-pelleted during precipitation, ethanol washing of DNA pellets was not sufficient, therefore it was necessary to include a further clean-up step, hence the sodium iodide method described by Li and Sheen (2012) was retained. The resuspended DNA were transferred to microfuge tubes, adding 2 vol 6 M sodium iodide and 100 μL 100 mg/mL silica dioxide. Tubes were foiled and placed on a tube rotator at low speed for 1 hr. at ambient temperature to facilitate DNA binding to the silica resin before pelleting samples by centrifugation, discarding the supernatant. Samples were then washed at least three times through resuspension in 500 μL DNA wash buffer (50% *v*/*v* ethanol, 10 mM Tris-HCl pH 7.5, 100 mM NaCl, 1 mM EDTA pH 8.4), followed by pelleting through centrifugation, removing the wash supernatant after pelleting. In samples where the silica matrix and wash supernatant were still muddy after three washes, wash steps were repeated until clear. After sufficient washing, samples were pelleted again by centrifugation, discarding the wash solution and incubated at 70°C for 1 min to evaporate residual ethanol. Thereafter 30 μL TE buffer was added to resuspend the silica matrix and incubated at 70°C for2 min to facilitate elution. Samples were then centrifuged (10,000 x *g)* at 4°C for 2 min, to pellet the silica matrix before transferring the supernatant containing isolated eDNA to a new microfuge tube and stored at −20°C until use.

### Extraction free environmental DNA isolation of water samples

Moat water collected from La Trobe University described earlier was used in developing a field useable water sampling method. Water samples were aliquoted into 50 mL conical tubes and spiked with the same quantities of *A. tomentosa* DNA detailed previously with starting DNA quantities of 1, 0.5, 0.25, and 0.125 μg added to each aliquot. Final concentrations of DNA were back calculated to for approximate quantities of 2 × 10^− 2^_,_ 1 × 10^− 2^_,_ 5 × 10^− 3^ and 2.5 × 10^− 3^ ng/μL per sample respectively. Larger volumes were also assessed, using 250 mL moat water aliquots whilst retaining the same starting quantities of *A. tomentosa* DNA as used in the 50 mL volumes. These samples were calculated with estimated final concentrations of 8.09 × 10^− 3^, 2.94 × 10^− 3^, 7.80 × 10^− 4^, and 3.95 × 10^− 4^ ng/μL each.

A negative control moat water with no spiked *A. tomentosa* was also included to assess non-target amplification. Samples were aspirated through a 50 mL Luer-Lok® syringe before attaching a 0.22 μM Sterivex® Millipore Express (PES) cartridge (Cat #: SVGP1050, Sigma). Samples were filtered until either the filter was clogged, or the full sample volume was processed, discarding the filtrate. To elute the DNA, the syringe and filter were reversed and back drawn to recover filter contents. The Sterivex® filter was discarded and contents transferred to a microfuge tube prior to amplification. Samples were diluted 1/10 consistent with Rathinasamy et al. (2018) for DNA amplification to minimise the likelihood of DNA amplification inhibition from organic DNA polymerase inhibitors commonly found in environmental samples.

### Data analysis

To obtain actual concentrations of DNA from artificially spiked water samples, the adjusted Cq values from the qPCR runs were exported from the MIC qPCR program after correcting the analysis method, fluorescence threshold and excluded cycles to Microsoft Excel (Version 2107, Build 14,228.20250). To calculate DNA quantity from each sample relative to their respective Cq values and estimated starting quantities, the equation y = mx + b was rearranged to x = ((y-b)/m), where x is the log value of DNA, y, the average Cq value from duplicate replicates, and b + m the slope and intercept being − 3.42 and 6.31 obtained from the qPCR standard curve with final concentrations adjusted to account for the 1/10 dilution factor used in this study (Stephenson 2010).

## Supplementary Information

**Additional file 1: Figure S1.** Assessing effect of increased inner primer concentrations to AtLAMP amplification times. A Inner primer concentrations were increased from 1.6 μM to 1.8 and 2 μM each FIP and BIP, assessed with a starting concentration of 5 × 10^− 1^ ng/μL *A. tomentosa* standards and B compared against a previous ten-fold serial dilution of *A. tomentosa* standards using 1.6 μM inner primers. Increased primer concentrations of 1.8 and 2 μM failed to decrease T_p_’s, therefore 1.6 μM was chosen as the optimal concentration of inner primers for AtLAMP.

## Data Availability

All data generated during this study are included in the published article.

## Abbreviations

DNA: Deoxyribonucleic acid
eDNA: Environmental deoxyribonucleic acid
LAMP: Loop-mediated isothermal amplification
qPCR: Quantitative polymerase chain reaction
