# Supplementary figures and images for: Development of a loop-mediated isothermal amplification assay for detection of Austropeplea tomentosa from environmental water samples
Source: Anim Dis. 2022 Dec 12;2(1):29. doi: 10.1186/s44149-022-00061-9 (PMC9743122; doi:10.1186/s44149-022-00061-9)

A

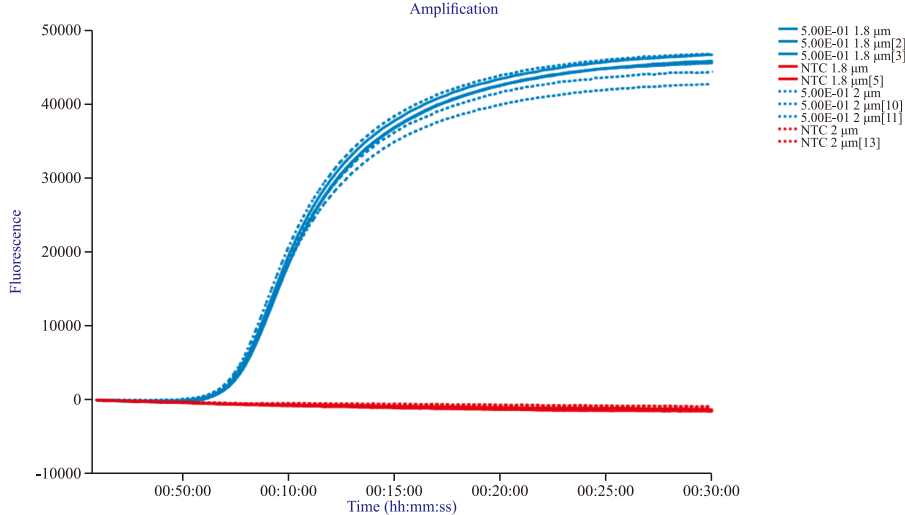

B

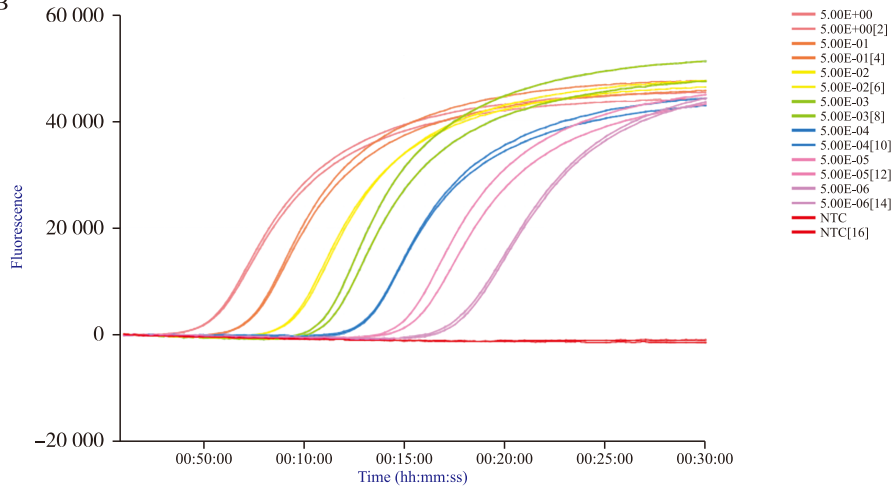

Supplement: Supplementary file 1 — Additional file 1: Figure S1. Assessing effect of increased inner primer concentrations to AtLAMP amplification times. A Inner primer concentrations were increased from 1.6 μM to 1.8 and 2 μM each FIP and BIP, assessed with a starting concentration of 5 × 10− 1 ng/μL A. tomentosa standards and B compared against a previous ten-fold serial dilution of A. tomentosa standards using 1.6 μM inner primers. Increased primer concentrations of 1.8 and 2 μM failed to decrease Tp’s, therefore 1.6 μM was chosen as the optimal concentration of inner primers for AtLAMP. [file 44149_2022_61_MOESM1_ESM.pdf]
